# Update to the study protocol for an implementation-effectiveness trial comparing two education strategies for improving the uptake of noninvasive ventilation in patients with severe COPD exacerbation

**DOI:** 10.1186/s13063-021-05855-9

**Published:** 2021-12-16

**Authors:** Mihaela S. Stefan, Penelope S. Pekow, Christopher M. Shea, Ashley M. Hughes, Nicholas S. Hill, Jay S. Steingrub, Mary Jo S. Farmer, Dean R. Hess, Karen L. Riska, Taylar A. Clark, Peter K. Lindenauer

**Affiliations:** 1 Institute for Healthcare Delivery and Population Science, University of Massachusetts Medical School, Baystate, Springfield, MA USA; 2 Department of Medicine, University of Massachusetts Medical School, Baystate, Springfield, MA USA; 3 School of Public Health and Health Sciences, University of Massachusetts, Amherst, MA USA; 4 Department of Health Policy and Management, Gillings School of Global Public Health, University of North Carolina-Chapel Hill, Chapel Hill, NC USA; 5 College of Applied Health Science at the University of Illinois at Chicago, Chicago, IL USA; 6 Division of Pulmonary and Critical Care Medicine, Tufts University School of Medicine, Boston, MA USA; 7 Division of Pulmonary and Critical Care, Department of Medicine, University of Massachusetts Medical School, Baystate, Springfield, MA USA; 8 Department of Population and Quantitative Health Sciences, University of Massachusetts Medical School, Worcester, MA USA; 9 College of Professional Studies, Respiratory Care Leadership, Northeastern University, Boston, MA USA; 10 Department of Respiratory Care, Massachusetts General Hospital, Boston, MA USA

## Abstract

**Background:**

There is strong evidence that noninvasive ventilation (NIV) improves the outcomes of patients hospitalized with severe COPD exacerbation, and NIV is recommended as the first-line therapy for these patients. Yet, several studies have demonstrated substantial variation in NIV use across hospitals, leading to preventable morbidity and mortality. In addition, prior studies suggested that efforts to increase NIV use in COPD need to account for the complex and interdisciplinary nature of NIV delivery and the need for team coordination. Therefore, our initial project aimed to compare two educational strategies: online education (OLE) and interprofessional education (IPE), which targets complex team-based care in NIV delivery. Due to the impact of the COVID-19 pandemic on recruitment and planned intervention, we had made several changes in the study design, statistical analysis, and implementation strategies delivery as outlined in the methods.

**Methods:**

We originally proposed a two-arm, pragmatic, cluster, randomized hybrid implementation-effectiveness trial comparing two education strategies to improve NIV uptake in patients with severe COPD exacerbation in 20 hospitals with a low baseline rate of NIV use. Due to logistical constrains and slow recruitment, we changed the study design to an opened cohort stepped-wedge design with three steps which will allow the institutions to enroll when they are ready to participate. Only the IPE strategy will be implemented, and the education will be provided in an online virtual format. Our primary outcome will be the hospital-level risk-standardized NIV proportion for the period post-IPE training, along with the change in rate from the period prior to training. Aim 1 will compare the change over time of NIV use among patients with COPD in the step-wedged design. Aim 2 will explore the mediators’ role (respiratory therapist autonomy and team functionality) on the relationship between the implementation strategies and effectiveness. Finally, in Aim 3, through interviews with providers, we will assess the acceptability and feasibility of the educational training.

**Conclusion:**

The changes in study design will result in several limitation. Most importantly, the hospitals in the three cohorts are not randomized as they enroll based on their readiness. Second, the delivery of the IPE is virtual, and it is not known if remote education is conducive to team building. However, this study will be among the first to test the impact of IPE in the inpatient setting carefully and may generalize to other interventions directed to seriously ill patients.

**Trial registration:**

ClinicalTrials.govNCT04206735. Registered on December 20, 2019;

## Update

This update pertains to the study design, recruitment, implementation strategies, and statistical analysis and should be read alongside the original publication [1]. The changes we employed are in response to the COVID-19 pandemic impact on recruitment and proposed intervention [2, 3]. All the changes in the protocol have been approved by the Baystate IRB and by the NHLBI.

## Study design revisions

The original study design was a cluster-randomized controlled 2-arm parallel trial, with 20 hospitals randomized to OLE or IPE. Due to logistical constraints and slow recruitment, we changed the design to an open cohort stepped-wedge design with three steps [4–6]. All participating hospitals will be assigned to the IPE strategy at one of three different time points (three steps at 5-month intervals). The hospitals will choose the step (time) to enroll. This significant change in design was determined by differences in institutions’ readiness for participation, which were in turn influenced by the local impact of COVID-19 infection and other priorities. Some institutions were ready to enroll in Spring 2021, while others wanted to delay enrollment to Fall 2021 or sometime in 2022. Based on their feedback, we understood that waiting several months after the institution signed the agreement could diminish their interest, their priorities could change, or their motivations for initially agreeing to adopt the program may no longer apply. Because we will enroll hospitals based on their readiness, randomization is not possible. Therefore, we decided to offer three start dates (May 2021, January 2022, and July2022) and enroll hospitals depending on their readiness to implement the intervention. This design accounts for the timing of the intervention, being particularly suited to the needs of hospital administrators. It avoids hospitals’ withdrawal or noncompliance, which could occur if they were assigned to a date they were not ready for.

## Hospital recruitment revisions

Initial recruitment began in January 2020 and recruitment was subsequently halted in April 2020 due to the global COVID-19 pandemic. We restarted recruitment in July 2020 and involved several strategies such as mailers, social media posts, and emails, which targeted chairs or department directors of nursing, respiratory therapy, critical care or pulmonology, and quality improvement officers. Despite all these strategies, recruitment was slow as providers’ priorities were focused on the pandemic. Nevertheless, seven hospitals expressed interest, and we had the first group training in May 2021. Thirty clinicians attended.

## Implementation strategies revisions

This trial was initially designed to compare two implementation strategies: one active control consisting of traditional, Online Education (OLE), and an in-person interactive Interprofessional Education (IPE) strategy. Moving forward, we will implement only the IPE strategy due to the change in the study design. Due to COVID-19 safety protocols, which limit travel and in-person meetings, the training is provided in an online virtual format. Due to the change in the study design, we will not have an active control (OLE).

We had planned to use a train-the-trainer model. Hospitals would send teams of Champions, one nurse, one respiratory therapist (RT), and one physician, to a 1-day in-person event, and this COPD-NIV Champion team would then train their colleagues. However, because of the COVID-19 global pandemic, we realized that the COPD-NIV Champion team would not have the capacity/time to be engaged at this level. Additionally, the implementation of new standards of practice that prohibited in-person meetings required a redesign of the IPE intervention, as described below.

Training of the champions was changed to an online virtual 1-day meeting. We recorded all the sessions so that participants would have ongoing access to the content. We created a home page on the online education platform (EthosCE®) for the COPD-NIV Champion team that includes recorded presentations, slides, algorithms, tip sheets, and landmark articles about NIV in COPD and teamwork.

### Delivery of the IPE at hospital level (for other clinicians) revisions

As in-person meetings were prohibited in most institutions, the training of the clinicians had to be changed to an online platform. We created two NIV-IPE clinician courses, a 90-min presentation for RTs and physicians, and a 60-min presentation for nurses, and posted these to the online educational platform. We employed this approach because we understood that champions would not have enough time to provide training to their colleagues, and clinicians need a flexible option to access the course at their own time and pace. The course was designed to cover clinician roles in the NIV delivery process, NIV knowledge, and the importance of teamwork and communication to improve patient outcomes.

## Statistical analysis revisions

### Aim 1

In the original submission, we had planned to compare the effectiveness of the OLE and IPE for increasing the delivery of NIV in appropriate patients hospitalized with COPD exacerbation. Due to the changes in the study to a stepped-wedge design, we have revised this aim to compare the effectiveness of IPE to standard care before the implementation of the IPE strategy. Our primary outcome remain the hospital-level risk-standardized (RS) NIV proportion however we will assess the change in rate from the period prior to training (not compared with the active control, OLE). Similar changes will apply to the secondary outcome measures of RS-hospital rates of NIV failure (invasive mechanical ventilation (IMV) after a trial of NIV), mortality, length of stay, and 30-day readmission among all patients with COPD. We did not change the time periods for comparison. We have planned three 18-month periods of analysis. (1) Baseline—18 months prior to the start; (2) immediate/short-term impact—18 months after start; and (3) sustainability—18 months after period 2. We have revised the original analysis by developing four risk-standardization models for the baseline 18 months, then moving forward 6 months and modeling an 18-month period. We will remove the four months after the IPE training for each step (cohort) from the analysis to allow for the completion of the educational sessions. This is a change from the original protocol based on the learning from the first cohort; it took on average 4 months for the champions to get the other providers enrolled in the educational activity. In a sensitivity analysis, we will repeat these analyses, using baseline data from the period prior to March 2020. We will compare RS-NIV rates from the pre-COVID-19 period to the “baseline” prior to implementation of IPE to gain some understanding of the changes in ventilation practice with COPD patients related to the COVID era.

### Patient and hospital information revisions

We will add the 7-day average of hospitalized patients with COVID-19 and the 7-day average of bed occupancy (adult inpatient and intensive care units) alongside the staffing: number of RTs, hospitalists, and emergency room physicians and nurses, to gain some understanding of the impact of COVID-19 pandemic. These factors will be used to describe participant hospitals.

### Statistical analysis Aim 1 revisions

With the new stepped-wedge study design, we will generate descriptive statistics overall, by hospital, and pre-and post-implementation, including counts and percentages for categorical data and means, standard deviations, and percentile distributions for continuous data. We will compare characteristics of hospitals started at each step, including size, ownership, teaching status, location, baseline NIV proportion, staffing of RTs, nurses, hospitalists, intensivists, and emergency room physicians via chi-square tests, and analysis of variance or Kruskal-Wallis tests.

Characteristics of eligible COPD patients derived from de-identified administrative and billing data of the enrolled hospitals will initially be compared via GEE models accounting for the pre- and post-implementation periods. Then, for each hospital, for each period, we will calculate the percentage of patients treated according to each of the primary ventilatory strategies: no assisted ventilation, NIV, and IMV. We will then calculate the percentage of patients initially treated with NIV among those who received assisted ventilation.

### Power and sample size for Aim 1 revisions

Originally, we had calculated that a total sample of 20 hospitals, 10 in each arm, will give 80% power to detect a difference of 15% in change (e.g., 5% increase among OLE hospitals, vs. 20% increase among IPE hospitals). For the stepped-wedge design, using a type I error rate of 0.05 and standard deviation of change in rates over time derived from our prior work with the Premier database, a total sample of 20 hospitals will give 80% power to detect a difference of 15% in change from baseline RS-NIV rates in hospitals before to the implementation to RS-NIV rates after the IPE education. However, to account for potential loss in recruited hospital sites, we aim to recruit up to 30 hospitals to achieve 80% power.

### Aim 2 analysis revision

As in our original submission, we plan to only examine the effect of the IPE education on RT autonomy and team functionality as potential mediators of NIV uptake.

### Statistical analysis revision

We will develop a series of models evaluating associations; however, instead of this being among the intervention (OLE and IPE), we will develop this for pre- and post-implementation and the mediators and outcome, including a structural equation model (SEM) to estimate the role of mediators as well as the direct effect of IPE education on the outcome.

### Power and sample size for Aim 2 revisions

The original study design power analysis for this aim accounted for the clustering of the 20 hospitals into their respective IPE and OLE arms. The revised design will have 20 hospitals in the IPE education. We calculate that to achieve 80% power and using a type I error rate of 0.05, a sample of 10 RT’s per hospital will allow us to detect a moderate (Cohen’s *d*=.4) difference at 1-year post-intervention. We will use an intraclass correlation (ICC) in the range of .10–.20.

### Aim 3 revisions

We plan to evaluate the strategies used and the barriers the COPD-NIV Champion teams faced to implement the NIV-IPE courses, to refine the implementation strategies further.

### Participant revisions

We will perform semi-structured interviews with the COPD-NIV Champions to assess the strategies they used and the barriers they faced when implementing the NIV-IPE courses, including the impact of the COVID-19 pandemic. We expect to enroll 2–3 providers from each hospital for a total of 14–21 Champions, enabling us to reach thematic saturation.

This qualitative aim will allow us to refine the intervention and the Champion’s role for each profession for future implementation strategies using an interprofessional team approach.

### Current status of the study

We have completed the training for the first cohort of seven hospitals and thirty champions. We are interviewing the champions to understand barriers and facilitators for engaging clinicians in the educational intervention. We have continued recruiting hospitals, and we have a pool of at least 23 hospitals interested to join the learning collaborative in 2022.

## Data Availability

Not applicable for this section.

## Abbreviations

COPD: Chronic obstructive pulmonary disease
IMV: Invasive mechanical ventilation
NIV: Noninvasive ventilation
RT: Respiratory therapist
OLE: Online education
IPE: Interprofessional education
COPD-NIV Champion Team: Chronic obstructive pulmonary disease-noninvasive ventilation champion team
RS: Risk standardized
GEE models: Generalized estimating equations model
